# Resistance to pyrethroids and the relationship between adult resistance and knockdown resistance (*kdr*) mutations in *Aedes albopictus* in dengue surveillance areas of Guizhou Province, China

**DOI:** 10.1038/s41598-024-63138-0

**Published:** 2024-05-28

**Authors:** Yan Zhang, Dan Wang, Weifang Shi, Jingzhu Zhou, Yulong Xiang, Yuwei Guan, Xuexue Kong, Wenqin Liang, Yong Hu

**Affiliations:** 1 Department of Vector Surveillance, Experimental Center, Guizhou Center for Disease Control and Prevention, Guiyang, 550004 Guizhou China; 2 School of Public Health, the Key Laboratory of Environmental Pollution Monitoring and Disease Control, Ministry of Education, Guizhou Medical University, Guiyang, 550025 Guizhou China

**Keywords:** *Ae. Albopictus*, *Kdr* mutation, Insecticide resistance, Dengue, Pyrethroid, Biochemistry, Molecular biology

## Abstract

The *Ae. albopictus* mosquito has gained global attention due to its ability to transmit viruses, including the dengue and zika. Mosquito control is the only effective way to manage dengue fever, as no effective treatments or vaccines are available. Insecticides are highly effective in controlling mosquito densities, which reduces the chances of virus transmission. However, *Ae. albopictus* has developed resistance to pyrethroids in several provinces in China. Pyrethroids target the voltage-gated sodium channel gene (*VGSC*), and mutations in this gene may result in knockdown resistance (*kdr*). Correlation studies between resistance and mutations can assist viruses in managing *Ae. albopictus*, which has not been studied in Guizhou province. Nine field populations of *Ae. albopictus* at the larval stage were collected from Guizhou Province in 2022 and reared to F1 to F2 generations. Resistance bioassays were conducted against permethrin, beta-cypermethrin, and deltamethrin for both larvae and adults of *Ae. albopictus*. *Kdr* mutations were characterized by PCR and sequencing. Additionally, the correlation between the *kdr* allele and pyrethroid resistance was analyzed. All nine populations of *Ae. albopictus* larvae and adults were found to be resistant to three pyrethroid insecticides. One *kdr* mutant allele at codon 1016, one at 1532 and three at 1534 were identified with frequencies of 13.86% (V1016G), 0.53% (I1532T), 58.02% (F1534S), 11.69% (F1534C), 0.06% (F1534L) and 0.99% (F1534P), respectively. Both V1016G and F1534S mutation mosquitoes were found in all populations. The *kdr* mutation F1534S was positively correlated with three pyrethroid resistance phenotypes (OR > 1, *P* < 0.05), V1016G with deltamethrin and beta-cypermethrin resistance (OR > 1, *P* < 0.05) and F1534C only with beta-cypermethrin resistance (OR > 1, *P* < 0.05). Current susceptibility status of wild populations of *Ae. albopictus* to insecticides and a higher frequency of *kdr* mutations from dengue-monitored areas in Guizhou Province are reported in this paper. Outcomes of this study can serve as data support for further research and development of effective insecticidal interventions against *Ae. albopictus* populations in Guizhou Province.

## Introduction

*Aedes albopictus* Skuse, 1895 (Diptera: Culicidae)^1^, also called the Asian tiger mosquito, is a widely distributed and acknowledged dangerous vector of many arboviruses, including chikungunya virus (CHIKV), dengue virus (DENV), yellow fever virus, and Zika virus (ZIKV)^2^. *Ae. albopictus* is highly adapted to different ecosystems and therefore tends to develop higher densities, which will exacerbate the spread of the virus. This species, especially in Guizhou province has been identified as the major vector of dengue. High densities of *Ae. albopictus* were monitored from 2016 to 2020, with MOIs ranging from 5.82 to 7.95 and sting indexes ranging from 11.69 to 17.01 mosquitoes/person · h^3^.

Controlling the density of vector insects significantly reduces the prevalence of diseases such as dengue fever because of the inadequacy of treatments and vaccines^4^. Chemical insecticides, including pyrethroids, carbamates and organophosphorus insecticides, are widely used to reduce the density of *Ae. albopictus* larvae and adults, preventing the transmission of arboviral diseases, especially during disease outbreaks in China^5–7^. Pyrethroids have been the most widely used insecticides in government campaigns and by citizens since the 1980s due to their low toxicity and high efficiency^6,8–10^. However, years of preference have led to resistance to pyrethroids in most provinces and cities in China^11–13^. Insecticides resistance poses a significant threat to mosquito control efforts, and there is a risk of its increasing. The Global Plan for Insecticide Resistance Management aims to prevent such incidents from occurring^14,15^. Understanding the insecticide-resistance and its molecular mechanism is crucial for the effective implementation of insecticides. However, the resistance level along with the mechanisms involved in *Ae. albopictus* population of Guizhou Province are poorly known.

*Ae. albopictus* populations have demonstrated multiple resistance mechanisms under sustained selection pressure from different insecticide species^16^, including target insensitivity, behavioral resistance, and metabolic detoxification. Pyrethroids affect voltage-gated sodium channels (*VGSC*), disrupting the transmission of electrical signals and causing immediate knockdown and eventual death of the mosquito^17^. Target insensitivity is caused by mutations in the insecticide target site, specifically in the *VGSC* gene^18^. These mutations alter the configuration of the site, weakening the binding of pyrethroid insecticides to the sodium channel, resulting in target resistance.

Amino acid substitutions in *VGSC* lead to resistance to pyrethroid insecticides, called knockdown resistance (*kdr*)^19^. *Kdr* mutations have been associated with pyrethroid resistance in *Anopheles sinensis Wiedemann, Anopheles gambiae, Culex pipiens pallens*, and *Aedes aegypti* for several decades^20–23^. Since 2011, the F1534C mutant allele was first reported by Kasai et al. in *Ae. albopictus* from Singapore^24^. Additionally, F1534S/L/R/W mutations have also been detected in *Ae. albopictus*^24–26^. Furthermore, I1532T and V1016G/I mutations have also been identified^26–29^. In addition, it has been confirmed that the F1534S allele is associated with pyrethroid resistance^25,30^. The mutant alleles V1016G, F1534S and F1534C have been shown to be associated with strong pyrethroid resistance phenotypes^9,29^. Moreover, several mutations acting synergistically may lead to an increased extent of resistance^16^. However, it is worth noting that many studies on *Ae. albopictus* *kdr* mutations have been conducted mainly in southern and coastal cities of China. Guizhou Province is an inland city located in southwest China. Although resistance to pyrethroids has been found in recent years, there have been few studies on *kdr* mutations.

*Ae. albopictus* is an invasive species with a distribution that covers large parts of China. Its range is gradually expanding from the southeast to the west and north regions^31^. The susceptible status of *Ae. albopictus* has been reported in many provinces throughout China^25,32,33^. The objectives of this study were to determine the insecticide resistance status of *Ae. albopictus* populations from dengue surveillance areas in Guizhou Province to pyrethroid insecticides (deltamethrin, beta-cypermethrin, permethrin) and the corresponding *kdr* mutation. Additionally, we analyzed the correlation between pyrethroid resistance and *kdr* mutations.

## Results

### Larval resistance bioassays 

Larval bioassays revealed varying degrees of resistance to the three pyrethroid insecticides (deltamethrin, permethrin, beta-cypermethrin) among nine populations of *Ae. albopictus* (RR_50_ ≥ 5) (Table 1). The LC50 values of laboratory sensitive strains of *Ae. albopictus* against the three above insecticides were 0.00045 mg/l, 0.00129 mg/l, and 0.00073 mg/l, respectively. The LC_50_ range was 0.007 mg/l (PZ)–0.053 mg/l (ZY), and RR_50_ was 15.56–117.78 after exposure to deltamethrin in all populations; 0.022 mg/l (PZ)-1.822 mg/l (XY), 17.05–1458.91 to permethrin; 0.005 mg/l (PZ)–0.045 mg/l (ZY), 6.85–61.64 to beta-cypermethrin LC_50_. All populations tested exhibited high resistance (RR_50_ ranging from 10.96 to 1458.91), except for the PZ population (RR_50_ = 6.85) to the three pyrethroid insecticides.

**Table 1:** Sensitivity of *Ae. albopictus* larvae to deltamethrin in nine different populations in Guizhou Province, China.

Population	Deltamethrin		Permethrin		Beta-cypermethrin	
	LC_50_ (95%CI) (mg/L)	RR_50_	LC_50_ (95%CI) (mg/L)	RR_50_	LC_50_ (95%CI) (mg/L)	RR_50_
CJ	0.010 (0.008, 0.012)	22.22	0.045 (0.031, 0.066)	34.88	0.008 (0.006, 0.009)	10.96
CS	0.012 (0.010, 0.015)	26.67	0.092 (0.072, 0.118)	71.32	0.010 (0.008, 0.011)	13.70
ZY	0.053 (0.041, 0.068)	117.78	0.468 (0.356, 0.615)	362.79	0.045 (0.037, 0.061)	61.64
GY	0.021 (0.017, 0.026)	46.67	0.559 (0.443, 0.706)	433.33	0.012 (0.011, 0.013)	16.44
LB	0.027 (0.023, 0.031)	60.00	0.229 (0.160, 0.331)	177.52	0.021 (0.019, 0.024)	28.77
XY	0.028 (0.023, 0.036)	62.22	1.882 (1.454, 2.319)	1458.91	0.040 (0.029, 0.052)	54.79
JS	0.018 (0.014, 0.022)	40.00	0.455 (0.339, 0.575)	352.71	0.011 (0.009, 0.013)	15.07
PZ	0.007 (0.005, 0.008)	15.56	0.022 (0.015, 0.030)	17.05	0.005 (0.004, 0.006)	6.85
BJ	0.010 (0.008, 0.011)	22.22	0.085 (0.068, 0.105)	65.89	0.010 (0.009, 0.011)	13.70

RR_50_ (resistance ratio): LC_50_ test population/LC_50_ laboratory-susceptible strain.

### Adult resistance bioassays 

**Figure 1:**
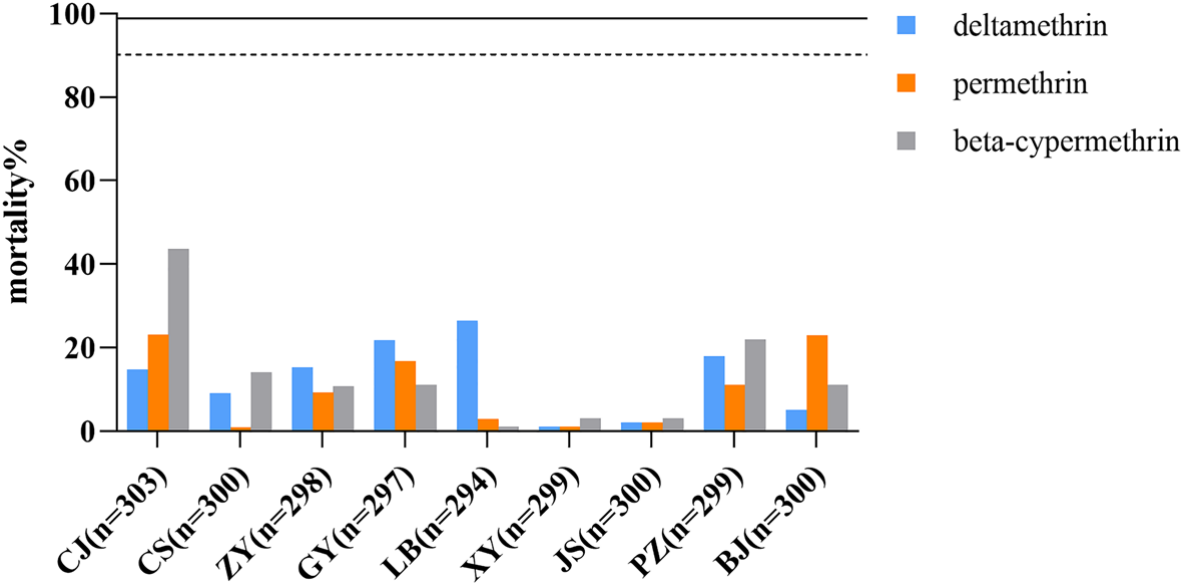
Mortality of *Ae. albopictus* field populations after 24 h exposure to three pyrethroids. Solid line represents mortality at 98%, dashed line as 90%.

Overall, all mosquito populations showed resistance to pyrethroid insecticides, with mortality rates ranging from 0.99 to 43.69% (Fig. 1). Among the three pyrethroid insecticides tested, *Ae. albopictus* exhibited higher resistance to permethrin (24-h mortality range 0.99–23.23%) than to deltamethrin (1.00–26.42%) and beta-cypermethrin (1.00–43.69%). The mortality rates for deltamethrin (1.00%, 2.00%), permethrin (1.01%, 2.00%), and beta-cypermethrin (3.00%, 3.00%) were much lower in the XY and JS populations of *Ae. albopictus*, indicating higher resistance.

### Frequency of *kdr* alleles and genotypes in field populations of *Ae. albopictus*

**Figure 2:**
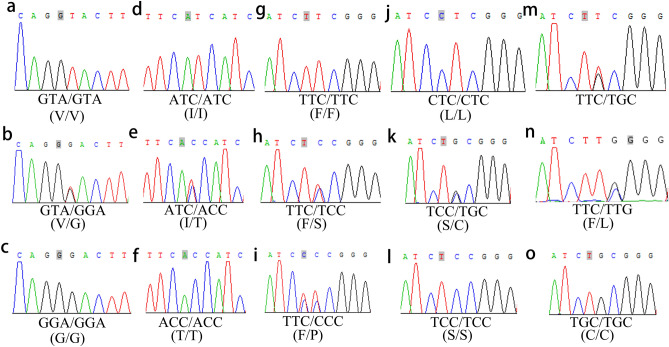
Sequence chromatograms of *Ae. albopictus* populations in nine dengue surveillance areas in Guizhou Province. (**a**–**c**) at locus 1016, (**d**–**f**) at locus 1532, and (**g**–**o**) at locus 1534.

Partial sequences of structural domains II and III of the *VGSC* gene were obtained from 2626 mosquitoes exposed to three pyrethroids. Non-synonymous *kdr* mutations were found at codon 1016 in domain II and codons 1532 and 1534 in domain III of the *VGSC* gene (Fig. 2). No synonymous mutations were recorded in this study.

**Figure 3:**
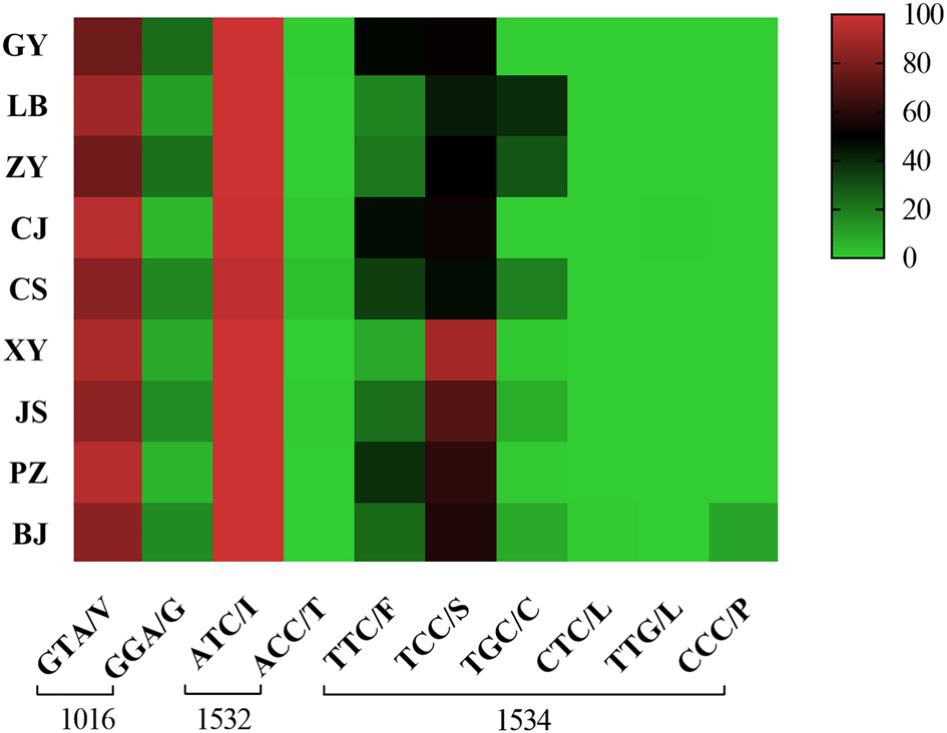
*Kdr* allele frequencies at loci 1016, 1532 and 1534 in field populations of *Ae. albopictus* in Guizhou Province, China**.**

**Figure 4:**
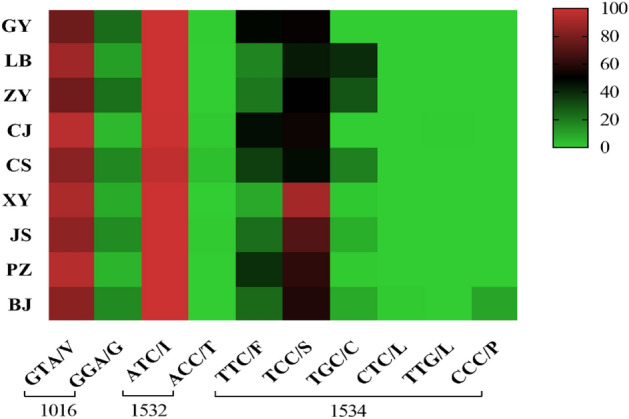
*Kdr* genotype frequencies at loci 1016, 1532 and 1534 in field populations of *Ae. albopictus* in Guizhou Province, China.

At codon 1016, two alleles were identified: the wild-type allele GTA/V and the mutant allele GGA/G (Table 2, Fig. 3). The allele V1016G was found in nine populations with a frequency of 13.86%, of which the highest frequency was 23.65% in GY. There existed a total of three genotypes as follows: wild-type genotype V/V (75.67%), wild-type mutant heterozygote V/G (20.94%) and mutant homozygous G/G (3.39%) (Table 3, Fig. 4). At codon 1532, the wild-type allele ATC/I (99.47%) was present in all populations, while the mutant allele ACC/T (0.53%) was only found in GY, CJ, CS, and JS populations (Table 2, Fig. 3). Three genotypes were identified: wild-type genotype I/I (99.20%), wild-type mutant heterozygote I/T (0.53%), and mutant homozygous T/T (0.27%) (Table 3, Fig. 4), of which the mutant genotype T/T was detected only in the CS population (2.37%). The study identified five mutant alleles at codon 1534, including TCC/S (58.02%), TGC/C (11.69%), CCC/P (0.99%), CTC/L (0.04%) and TTG/L (0.02%), in addition to the wild-type allele TTC/F (29.25%) (Table 2, Fig. 3). The nine populations revealed a total of nine genotypes: wild-type genotype F/F (13.10%), wild-type mutant heterozygotes F/S (28.87%), F/L (0.04%), F/C (1.41%), and F/P (1.98%), mutant genotypes S/S (41.55%), L/L (0.04%), and C/C (8.95%), and mutant heterozygotes S/C (4.07%) (Table 3, Fig. 4).

**Table 2:** *Kdr* allele mutation frequencies at loci 1016, 1532 and 1534 in the field population of *Ae. albopictus* in Guizhou Province, China.

Population	N	*kdr* allele									
		1016		1532		1534					
		GTA/V	GGA/G	ATC/I	ACC/T	TTC/F	TCC/S	TGC/C	CTC/L	TTG/L	CCC/P
GY	296	452	140	591	1	287	305	0	0	0	0
		(76.35)	(23.65)	(99.83)	(0.17)	(48.48)	(51.52)	(0.00)	(0.00)	(0.00)	(0.00)
LB	298	530	66	596	0	104	259	233	0	0	0
		(88.93)	(11.07)	(100.00)	(0.00)	(17.45)	(43.46)	(39.09)	(0.00)	(0.00)	(0.00)
ZY	294	455	133	588	0	123	292	173	0	0	0
		(77.38)	(22.62)	(100.00)	(0.00)	(20.92)	(49.66)	(29.42)	(0.00)	(0.00)	(0.00)
CJ	301	572	30	597	5	281	320	0	0	1	0
		(95.02)	(4.98)	(99.17)	(0.83)	(46.68)	(53.16)	(0.00)	(0.00)	(0.17)	(0.00)
CS	295	492	98	570	20	203	277	110	0	0	0
		(83.39)	(16.61)	(96.61)	(3.39)	(34.41)	(46.95)	(18.64)	(0.00)	(0.00)	(0.00)
XY	289	530	48	578	0	51	520	7	0	0	0
		(91.7)	(8.30)	(100.00)	(0.00)	(8.82)	(89.97)	(1.21)	(0.00)	(0.00)	(0.00)
JS	296	499	93	590	2	136	414	42	0	0	0
		(84.29)	(15.71)	(99.66)	(0.34)	(22.97)	(69.93)	(7.09)	(0.00)	(0.00)	(0.00)
PZ	288	543	33	576	0	222	350	4	0	0	0
		(94.27)	(5.73)	(100.00)	(0.00)	(38.54)	(60.76)	(0.69)	(0.00)	(0.00)	(0.00)
BJ	269	451	87	538	0	129	310	45	2	0	52
		(83.83)	(16.71)	(100.00)	(0.00)	(23.98)	(57.62)	(8.36)	(0.37)	(0.00)	(9.67)
Total	2626	4524	728	5224	28	1536	3047	614	2	1	52
		(86.14)	(13.86)	(99.47)	(0.53)	(29.25)	(58.02)	(11.69)	(0.04)	(0.02)	(0.99)

N is the sample number. Data outside parentheses are the number of individuals; data within parentheses are their frequency (%).

**Table 3:** *Kdr* genotype frequencies of mutations at loci 1016, 1532 and 1534 in field populations of *Ae. albopictus* in Guizhou Province, China.

Population	N	1016			1532			1534								
		V/V	V/G	G/G	I/I	I/T	T/T	F/F	F/S	F/L	F/C	F/P	S/S	L/L	C/C	S/C
GY	296	174	104	18	295	1		24	239				33			
		(58.78)	(35.14)	(6.08)	(99.66)	(0.34)		(8.11)	(80.74)				(11.15)			
LB		237	56	5	298			44	14		2		122		115	1
		(79.53)	(18.79)	(1.68)	(100.00)			(14.77)	(4.70)		(0.67)		(40.94)		(38.59)	(0.34)
ZY	294	200	55	39	294			18	56		31		73		26	90
		(68.03)	(18.71)	(13.27)	(100.00)			(6.12)	(19.05)		(10.54)		(24.83)		(8.84)	(30.61)
CJ	301	272	28	1	296	5		71	138	1			91			
		(90.37)	(9.30)	(0.33)	(98.34)	(1.66)		(23.59)	(45.85)	(0.33)			(30.23)			
CS	295	201	90	4	282	6	7	71	61				108		55	
		(68.14)	(30.51)	(1.36)	(95.59)	(2.03)	(2.37)	(24.07)	(20.68)				(36.61)		(18.64)	
XY	289	246	38	5	289			3	45				234			7
		(85.12)	(13.15)	(1.73)	(100.00)			(1.04)	(15.57)				(80.97)			(2.42)
JS	296	210	79	7	294	2		41	54				180		21	
		(70.95)	(26.69)	(2.36)	(99.32)	(0.68)		(13.85)	(18.24)				(60.81)		(7.09)	
PZ	288	255	33		288			41	140				103			4
		(88.54)	(11.46)		(100.00)			(14.24)	(48.61)				(35.76)			(1.39)
BJ	269	192	67	10	269			31	11		4	52	147	1	18	5
		(71.38)	(24.91)	(3.72)	(100.00)			(11.52)	(4.09)		(1.49)	(19.33)	(54.65)	(0.37)	(6.69)	(1.86)
Total	2626	1987	550	89	2605	14	7	344	758	1	37	52	1091	1	235	107
		(75.67)	(20.94)	(3.39)	(99.20)	(0.53)	(0.27)	(13.10)	(28.87)	(0.04)	(1.41)	(1.98)	(41.55)	(0.04)	(8.95)	(4.07)

N indicates the number of samples. Data outside parentheses are the number of individuals; data within parentheses are their frequency (%); no data are unlabeled to avoid clutter.

At codon 1534, the mutant allele TCC/S was detected in all populations, with mutation frequencies ranging from 43.46 to 89.97%. Only the BJ population had CTC/L and CCC/P, while TTG/L was exclusively found in the CJ population. The mutant genotype S/S was detected in all populations and was the dominant genotype in LB, ZY, CS, XY, JS and BJ populations with mutation frequencies of 40.94% (122/298), 24.83% (73/294), 36.61% (108/295), 80.97% (234/289), 60.81% (180/296), and 54.65% (147/269), respectively. Mutant homozygotes (C/C) were present in all five populations, with the highest frequency of 38.59% in LB. Mutant heterozygotes (S/C) were also present in all five populations, but not the same, with the highest frequency of 30.61% in ZY.

### Frequency and distribution of genotype combinations at loci 1016, 1532 and 1534 in *Ae. albopictus*

In all populations (2626 individuals in total), we found that some mosquito individuals carried *kdr* mutations in two codons together, and a total of 23 genotype combinations were identified, with no individuals carrying simultaneous mutations in all three loci (Table 4). Except for the GY and XY populations, one wild type (V/V + I/I + F/F) was present in all populations, with the highest frequency observed in the CJ population (18.27%). Twelve single mutant types were observed, including V/V + I/I + S/S and V/V + I/I + F/S, which were the most widespread (found in 9 populations) with frequencies of occurrence of 41.36% and 15.73%, respectively. Additionally, there were ten double mutant phenotypes, with the highest frequency of V/G + I/I + F/S mutations (12.83%), also found in 9 populations, and the highest frequency of 33.11% in GY.

**Table 4:** Genotypes and frequencies of three locus combinations of the *kdr* gene in the *Ae. albopictus* field population.

Type	BJ	CJ	CS	JS	LB	PZ	GY	XY	ZY	Total
V/V + I/I + F/F	1 (0.37)	55 (18.27)	21 (7.12)	4 (1.35)	1 (0.34)	28 (9.72)			2 (0.68)	112 (4.27)
V/G + I/I + F/F	21 (7.81)	13 (4.32)	36 (12.20)	29 (9.80)	39 (13.09)	13 (4.51)	6 (2.03)		4 (1.36)	161 (6.13)
G/G + I/I + F/F	9 (3.35)	1 (0.33)	4 (1.36)	7 (2.36)	4 (1.34)		18 (6.08)	3 (1.04)	12 (4.08)	58 (2.21)
V/V + I/T + F/F		2 (0.66)								2 (0.08)
V/V + T/T + F/F			7 (2.37)							7 (0.27)
V/V + I/I + F/C	3 (1.12)								3 (1.02)	6 (0.23)
V/V + I/I + F/S	5 (1.86)	120 (39.87)	8 (2.71)	5 (1.69)	1 (0.34)	120 (41.67)	140 (47.30)	7 (2.42)	7 (2.38)	413 (15.73)
V/V + I/I + C/C	18 (6.69)		55 (18.64)	20 (6.76)	114 (38.26)				25 (8.50)	232 (8.83)
V/V + I/I + S/C	5 (1.86)				1 (0.34)	4 (1.39)		7 (2.42)	90 (30.61)	107 (4.07)
V/V + I/I + S/S	147 (54.65)	91 (30.23)	107 (36.27)	180 (60.81)	120 (40.27)	103 (35.76)	33 (11.15)	232 (80.28)	73 (24.83)	1086 (41.36)
V/V + I/I + F/P	12 (4.46)									12 (0.46)
V/V + I/I + L/L	1 (0.37)									1 (0.04)
V/V + I/I + F/L		1 (0.33)								1 (0.04)
V/G + I/T + F/F			3 (1.02)	1 (0.34)						4 (0.15)
V/V + I/T + F/S		3 (1.00)	3 (1.02)	1 (0.34)			1 (0.34)			8 (0.30)
V/G + I/I + F/S	6 (2.23)	15 (4.98)	50 (16.95)	48 (16.22)	14 (4.70)	20 (6.94)	98 (33.11)	37 (12.80)	49 (16.67)	337 (12.83)
V/G + I/I + F/C									1 (0.34)	1 (0.04)
V/G + I/I + S/S			1 (0.34)		2 (0.67)			1 (0.35)		4 (0.15)
V/G + I/I + C/C				1 (0.34)	1 (0.34)				1 (0.34)	3 (0.11)
G/G + I/I + F/S								1 (0.35)		1 (0.04)
G/G + I/I + S/S								1 (0.35)		1 (0.04)
V/G + I/I + F/P	40 (14.87)									40 (1.52)
G/G + I/I + F/C	1 (0.37)				1 (0.34)				27 (9.18)	29 (1.10)

Data outside parentheses are the number of individuals; data within parentheses are their frequency (%); no data are unlabeled to avoid clutter.

### Correlation between *kdr* mutant genes and resistance phenotypes

The OR values and 95% CIs were calculated for the six kdr mutant alleles at loci 1016, 1532 and 1534 in *Ae. albopictus* exposed to deltamethrin, permethrin and beta-cypermethrin. The frequency of mutations (GGA/G) at codon 1016 was 9.29% in susceptible individuals and 14.78% in resistant individuals after exposure to deltamethrin. The OR was 1.69 (95% CI 1.06–2.71) and *P* < 0.05 (Table 5). The study found that in the *Ae. albopictus* population, the OR values were 1.33 (95% CI 0.83–2.11) (*P* > 0.05) and 2.21 (95% CI 1.33–3.70) (*P* < 0.05) against permethrin and beta-cypermethrin, respectively. Generally, the V1016G mutant allele was positively correlated with deltamethrin and beta-cypermethrin resistance genotypes. No statistical correlation (*P* > 0.05) was revealed between the I1532T mutant allele and the three pyrethroid resistance phenotypes at locus 1532. At codon 1534, the OR values were 1.95 (*P* < 0.05), 1.75 (*P* < 0.05), and 2.90 (*P* < 0.05) in the *Ae. albopictus* populations to deltamethrin, permethrin, and beta-cypermethrin, respectively (95% CI 1.44–2.65, 1.28–2.41, and 2.17–3.88, respectively), reflecting that the F1534S mutant allele was positively associated with the three pyrethroid resistance phenotypes. The association between the F1534C mutant allele and beta-cypermethrin insecticide resistance phenotype was positive (OR 5.57, 95% CI 3.01–10.31, *P* < 0.05). On the other hand, no statistically significant difference was observed between the F1534C mutant allele and deltamethrin and permethrin insecticide resistance phenotypes (*P* > 0.05). There were no statistically significant differences (*P* > 0.05) between the F1534P and F1534L mutant alleles and the resistance phenotypes after exposure to deltamethrin, permethrin, and beta-cypermethrin, respectively. However, a positive correlation was found between F1534P and the total pyrethroid resistance phenotype.

**Table 5:** Relationship between *kdr* gene mutation and insecticide resistance in the field population of *Ae. Albopictus.*

Insecticides	Type	N	*kdr* allele										OR (95% CI)					
			GTA/V	GGA/G	ATC/I	ACC/T	TTC/F	TCC/S	CTC/L	TTG/L	TGC/C	CCC/P	V1016G	11532 T	F1534S	F1534C	F1534P	F1534L
Deltamethrin	R	758	1292	224	1507	9	437	898			157	24	1.69*	0.99	1.95*	0.98	5.11	
			(85.22)	(14.78)	(99.41)	(0.59)	(28.83)	(59.23)			(10.36)	(1.58)	(1.06,2.71)	(0.99,1.00)	(1.44,2.65)	(0.64,1.52)	(0.68,38.23)	
	S	113	205	21	226		93	98			34	1						
			(90.71)	(9.29)	(100.00)		(41.15)	(43.36)			(15.04)	(0.44)						
Permethrin	R	777	1335	219	1549	5	417	946	2		177	12	1.33	1.00	1.75*	1.58	0.97	1.00
			(85.91)	(14.09)	(99.68)	(0.32)	(26.83)	(60.88)	(0.13)		(11.39)	(0.77)	(0.83,2.11)	(0.99,1.00)	(1.28,2.41)	(0.94,2.63)	(0.96,0.99)	(0.99,1.00)
	S	100	178	22	200		78	101			21	0						
			(89.00)	(11.00)	(100.00)		(39.00)	(50.50)			(10.50)	(0.00)						
Beta-cypermethrin	R	761	1297	225	1510	12	389	906			213	14	2.21*	0.92	2.90*	5.57*	4.39	1.01
			(85.22)	(14.78)	(99.21)	(0.79)	(25.56)	(59.53)			(13.99)	(0.43)	(1.33,3.70)	(0.21,4.15)	(2.17,3.88)	(3.01,10.31)	(0.57,33.73)	(0.99,1.02)
	S	117	217	17	232	2	122	98		1	12	1						
			(92.74)	(7.26)	(99.15)	(0.85)	(52.14)	(41.88)		(0.43)	(5.13)	(0.43)						
Total	R	2296	3924	668	4566	26	1243	2750	2		547	50	1.70*	1.87	2.18*	1.92*	5.89*	0.47
			(85.45)	(14.55)	(99.43)	(0.57)	(27.07)	(59.89)	(0.04)		(11.91)	(1.09)	(1.29,2.25)	(0.44,7.91)	(1.83,2.60)	(1.45,2.56)	(1.43,24.36)	(0.04,5.22)
	S	330	600	60	658	2	293	297	0	1	67	2						
			(90.91)	(9.09)	(99.70)	(0.30)	(44.39)	(45.00)	(0.00)	(0.15)	(10.15)	(0.30)						

N represents the sample number. S means susceptible phenotype: R stands for resistant phenotype. Data outside brackets indicate a number of individuals; data within brackets indicate the frequency (%); no data are unlabeled to avoid clutter.

*P < 0.05.

To explore whether the simultaneous presence of mutations at multiple loci in the one individual was the result of insecticide pressure, we classified the combinatorial genotypes into three categories: 0 (representing no mutations at loci 1016, 1532, and 1534), 1 (mutations at only one of the three loci), and 2 (mutations at only two of the three loci), and used chi-square tests to explore whether the combinatorial genotypes varied for different resistance phenotypes. The results showed significant differences (*P* < 0.05) in the types of genotypic combinations of different resistance phenotypes under the pressure of the three pyrethroid insecticides. The results of the comparison between the two types are summarized in Table 6.

**Table 6:** Genotypic combination type comparisons of different resistance phenotypes of *Ae. albopictus* under the pressure of three pyrethroid insecticides.

Type	Beta-cypermethrin		Permethrin		Deltamethrin		χ_1_^2^	χ_2_^2^	χ_3_^2^
	R	S	R	S	R	S			
0	5	24	27	14	24	18	101.67*	19.24*	29.06*
1	615	87	630	78	591	86			
0	5	24	27	14	24	18	104.48*	21.34*	35.35*
2	141	5	120	8	143	10			
1	615	87	630	78	591	86	10.05*	2.67	4.64
2	141	5	120	8	143	10			

S, susceptible phenotype; R, stands for resistant phenotype; χ_1_^2^, Value for beta-cypermethrin; χ_2_^2^, Value for permethrin; χ_3_^2^, Value for deltamethrin.

*p < 0.01.

## Discussion

In the study, nine field populations of *Ae. albopictus* were obtained from dengue surveillance areas in Guizhou Province, including surrounding and central areas, with 2,626 mosquitoes, the largest number in the same study to date. The results showed that *Ae. albopictus* (larvae and adult mosquitoes) of Guizhou had already accumulated serious resistance to pyrethroid insecticides (deltamethrin, permethrin, and beta-cypermethrin), approaching or exceeding the results of southern and coastal provinces and cities such as Zhejiang and Hainan in China^9,25,34^. In addition, molecular experiments showed that corresponding *kdr* mutations were identified in nine *Ae. albopictus* populations. The mutant alleles GGA/G at loci 1016, ACC/T at loci 1532, TTC/S, CTC/L, TTG/L, TGC/C and CCC/P at loci 1534 were detected in *Ae. albopictus* with the mutation frequencies 13.86%, 0.53%, 58.02%, 0.04%, 0.02%, 11.69%, and 0.99%, respectively. Correlation analyses of mutations in codons 1016, 1532 and 1534 with resistance phenotypes revealed that the V1016G mutation was significantly associated with *Ae. albopictus* resistance phenotypes against deltamethrin (OR > 1, *P* < 0. 05) and beta-cypermethrin (OR > 1, *P* < 0.05), while the F1534C mutation was positively correlated with beta-cypermethrin (OR > 1, *P* < 0.05) and the F1534S mutation was positively correlated for resistance to deltamethrin, permethrin and beta-cypermethrin (OR > 1, *P* < 0.05). However, no correlation was identified between the I1532T mutation and the three pyrethroid resistance phenotypes, which may be due to the low frequency of the mutation. The difference between the appearance of the *kdr* mutation and the absence of the mutation was significant (*P* < 0.0167) for all three insecticides, whereas the occurrence of one locus mutation and the occurrence of two showed a significant difference only in the case of beta-cypermethrin, suggesting that the difference between the resistant phenotype and the susceptible phenotype is mainly the production of the *kdr* mutation, and that simultaneous mutation of multiple loci may be the result of enhanced resistance.

In the absence of reports on the existence of *Aedes aegypti* in Guizhou currently, *Ae. albopictus* is the dominant vector for the transmission of dengue virus. The WHO recommended the global promotion of the use of pyrethroid insecticides for mosquito control in the late 1980s^35^. Since 1992, pyrethroid insecticides have been widely available because of their affordability, rapid knockdown, low toxicity to mammals and relative safety to humans^36^. We have reported pyrethroid resistance in wild populations of *Ae. albopictus* in 2018 and 2021, having observed susceptibility to pyrethroids in GY, CS and XY populations. In this study, bioassays showed increased resistance to pyrethroids in *Ae. albopictus* compared to the GY population in 2018^37^, and little more or slightly increased resistance in the CS and XY populations in 2021^38^. During our interventions, we noticed that householders in residential areas habitually spray commercially purchased insecticides around flowers and plants, which are complex insecticides containing several pyrethroids. The literature reports that 23 types of pyrethroid active ingredients are approved by the government of the People's Republic of China as public health insecticides^11^. Second, several cities, including ZY, CS and XY, had contracted with PCOs for regular insecticide spraying to reduce mosquito densities and breeding as part of their hygiene city campaigns. In these habitats, mosquitoes may have developed high levels of resistance at the larval and adult stages under the selective pressure of continuous exposure to pyrethroid insecticides. In addition, the XY and JS populations of *Ae. albopictus* showed lower mortality rates after exposure to the three pyrethroids than other populations, which may explain the difference in geographical location. In recent years, resistance of *Ae. albopictus* to pyrethroid insecticides has also been reported in other provinces and cities in China^2,39,40^, but the differences should be carefully analyzed as more factors affect the results of the bioassay, including the rearing sites, the samples tested and the assessment of mortality standards^9^. The results of this study enriched the information on the resistance of *Ae. albopictus* to pyrethroids in the dengue surveillance areas of Guizhou Province, providing more direct supporting data to guide the utilization of insecticides.

In the current study, the individuals we used for *kdr* gene testing were mosquitoes after bioassay, facilitating the accurate determination of the association between the mutation and pyrethroid resistance. We identified seven mutant alleles at codons 1016 (13.86%), 1532 (0.53%) and 1534 (70.75%) and further confirmed the correlation of F1534S with pyrethroids, which is consistent with the results of population studies in several geographical regions of China^9,25,34^. Second, in the face of the emergence of outbreaks in surrounding provinces and the existence of imported Dengue instances^41–44^, the higher mutation frequency may represent a serious threat to the control of potential Dengue outbreaks, even if there has been no indigenous Dengue outbreak at present in Guizhou Province. Moreover, the present study identified the V1016G mutation as positively associated with the resistance phenotype of *Ae. albopictus* to deltamethrin and permethrin, which has been demonstrated to be responsible for the insensitivity against these insecticides in reports of *Aedes Aegypti* but has hardly been reported in studies of *Ae. albopictus*. Some studies have documented a positive correlation between the I1532T mutation and the deltamethrin resistance phenotype^45^, but there are also conflicting views^9,34^. The I1532T mutation has a low mutation frequency and was not found to be associated with its insecticide resistance in the present results, which needs to be further investigated. Our results showed that F1534C was only insensitive to beta-cypermethrin. Although a resistance relationship between F1534C and pyrethroids has been detected in *Aedes aegypti*^46^, few correlations have been reported in *Ae. albopictus* in China^9,13,33^. F1534C could be a weaker indicator of resistance. Significantly, a new mutation, F1534P, was identified in the current study, but it was only found in one population, and its association with resistance needs to be further investigated.

A total of 29 different genotypic combinations were observed at codons 1016, 1532 and 1534. Among these, the F1534S single mutation at three loci was the most common combination, distributed across all populations, corresponding to the findings of Zhao et al. on field populations of *Ae. albopictus* in 11 dengue-endemic provinces^12^. The interactions between mutations at different loci remain to be further investigated. Differences in mutation rates at different loci in different geographical regions may result from uneven distribution of resistance^39^ and the presence of multiple resistance mechanisms, resulting in the *kdr* mutation not playing a dominant role. Further investigation of other resistance mechanisms, such as metabolic resistance^1,47^, should also be conducted. In addition, Chen and Zhao et al. found that the mutation rates at loci 1016, 1532, and 1534 were correlated with average annual temperature, rainfall, and dengue-endemic areas^12,48^. Therefore, monitoring the *kdr* mutation frequency is not only conducive to the monitoring of *Ae. albopictus* resistance to pyrethroids and controlling dengue vector density, but also to preventing dengue outbreaks in advance.

## Conclusion

Current susceptibility status of wild populations of *Ae. albopictus* to insecticides and a higher frequency of *kdr* mutations from dengue-monitored areas in Guizhou Province are reported in this paper. The pyrethroid resistance phenotype was clearly associated with the *kdr* mutation F1534S, which can be considered a molecular marker for resistance. In addition, outcomes of this study can serve as data support for further research and development of effective insecticidal interventions against *Ae. albopictus* populations in Guizhou Province.

## Materials and methods

### Collection of wild *Ae. albopictus* samples

Nine field populations of *Ae. albopictus* were collected using Pasteur pipettes from breeding sites, such as flowerpots, waste tires, metal containers, waste buckets, water tanks and other water containers from Guiyang (GY), Ziyun (ZY), Jinsha (JS), Libo (LB), Xingyi (XY), Bijiang (BJ), Congjiang (CJ), Panzhou (PZ) and Chishui (CS) in Guizhou Province during July–August 2022. The larvae were identified as *Ae. albopictus* species by using the taxonomic keys^49^. All the samples collected in the field in each region were brought back to the laboratory of the Guizhou Provincial Centre for Disease Control and Prevention in buckets and identified again, and finally the selected *Ae. albopictus* mosquitoes were reared in tubs filled with dechlorinated water. The larvae were reared to adulthood under standard conditions of 26 ± 1 °C, 75 ± 5% relative humidity and 14/10 h light/dark photoperiod. Larvae were fed a special powdered diet (pig liver: steamed bread = 1:1) and adults were fed a 10% sucrose solution. All *Ae. albopictus* directly collected and identified from the field (F0 generation) were reproduced (F1–F2 generations) before being tested for susceptibility.

The susceptible strains of *Ae. albopictus* which served as a reference, was kindly provided by National Institute for Communicable Disease Control and Prevention, Chinese Center for Disease Control and Prevention. The strain has been maintained in the insectary over a decade, without exposure to any insecticide.

### Larval resistance bioassays

The susceptibility of larvae was determined using three insecticides (permethrin 90% pure, deltamethrin 98%, beta-cypermethrin 91.16%) from the Chinese Center for Disease Control and Prevention, complying with WHO guidelines^50^. Firstly, all insecticides were diluted to 5–7 concentrations with acetone to obtain mortality from 10 to 90%. Then, 1 ml diluted insecticide solution and 20–30 late third and fourth instar larvae were added to an experimental beaker that held 199 ml water separately. Furthermore, insecticide solution was replaced by acetone in the control group. Larval of *Ae. albopictus* mortalities were recorded after 24 h exposure. Larvae that did not move or twitched when strongly stimulated were considered dead. The assay was repeated three times. Larval mortality was calculated by dividing the number of dead larvae by the total number tested. The extent of resistance is defined by the resistance ratio (RR_50_), which was derived by comparing the median lethal concentration (LC_50_) value of the field population with the LC_50_ value of the sensitive laboratory population to the insecticide.

### Adult resistance bioassays

The susceptibility of adults was tested using three insecticide-impregnated papers (0.4% permethrin, 0.03% deltamethrin, 0.08% beta-cypermethrin) from the Chinese Center for Disease Control and Prevention complying with WHO guidelines56^51^. In the assay, 25 female mosquitoes, non-blood-fed, aged 3–5 days were selected to the resting tube without insecticide to adaptation for 30 min. After that they were gently blown into experimental tubes containing insecticide impregnated paper for up for 1 h. Silicone oil-treated papers without insecticides were applied to the control groups. Following insecticide exposure, mosquitoes were returned to resting tubes with 10% sugar water and mortality assessed after 24 h. Approximately 100 female mosquitoes were tested for each insecticide. Mosquitoes that do not move or only tremble in their limbs and wings and cannot survive are considered dead. They are also considered susceptible phenotypes. In contrast, mosquitoes that can survive are considered resistant phenotypes. Dead and survival mosquitoes were collected individually in test tubes filled with 95% alcohol at − 80 °C for subsequent DNA analysis. The bioassay should be repeated if the mortality in the control group was ≥ 20%. The test group mortality rate would be adjusted using Abbott's formula if the control group mortality rate was between 5 and 20%: Corrected mortality (%) = (test group mortality − control group mortality)/(1 − control group mortality) × 100^51^.

### DNA extraction and mutation detection

Ezup Columnar Animal Tissue Genomic DNA Extraction Kit (Sangon Biotech, B518251, shanghai) was used to extract genomic DNA from a single mosquito for identification of the *kdr* mutations. Partial domain II and III of the voltage-gated sodium channel gene (*VGSC*) (covering I1532, F1534 and V1016) were amplified using the primer aegSCF20 (forward: 5′-GAC AAT GTG GAT CGC TTC CC-3′) and aegSCR21 (reverse: 5′-GCA ATC TGG CTT GTT AAC TTG-3′), aegSCF7(forward: 5′-GAG AAC TCG CCG ATG AAC TT-3′) and aegSCR7 (reverse: 5′-GAC GAC GAA ATC GAA CAG GT-3′). The PCR cycling parameter includes the following: pre-denaturation at 95 °C for 5 min, then ten cycles of 94 °C for 30 s, 63 °C for 30 s and 72 °C for 30 s, and followed by 30 cycles of 95 °C for 30 s, 58 °C for 30 s and 72 °C for 30 s, ending with an extension at 72 °C for 10 min. After electrophoresis, PCR products were purified using San Prep Column-based DNA Gel Recovery Kit (Sangon, Shanghai) and sequenced reversely with the primer aegSCR22 (5′-TTC ACG AAC TTG AGC GCG TTG-3′) and aegSCR8 (5′-TAG CTT TCA GCG GCT TCT TC-3′) respectively using the ABI 3730XL automatic sequencer (Applied Biosystems, Shanghai, China). Sequencing was accomplished by Sangon Biotech (Shanghai) Co., Ltd. All sequenced data were analyzed with sequence analysis software.

### Statistical analysis

The resistance status of adult mosquitoes was also classified according to WHO criteria^51^: a mortality < 90% was recognized as resistant, a mortality of 90–98% as probable resistance, and a mortality > 98% was susceptible. For larval bioassays, susceptible if RR_50_ < 5, moderately resistant if 5 < RR_50_ ≤ 10, and highly resistant if RR_50_ > 10. A chi-squared test and hazard analysis were used to analyze the correlation between the frequency of mutant alleles and their resistance phenotypes, and the dominance ratio (OR) values were calculated. Differences were determined to be statistically significant at *P* < 0.05; the relationship between the *kdr* allele and the resistance phenotype was considered positive when the OR > 1 and negative when the OR < 1, and not yet statistically significant or statistically insignificant if the 95% CI of the OR value ranged between 1 or a *P* > 0.05. All data were analysed using excel2013 and spss24.0 software.

## Data Availability

Data underlying the conclusions are presented in the article. The datasets used and analyzed during this study are available from the corresponding author upon reasonable request.

## References

[1] Rahman RU (2021). Insecticide resistance and underlying targets-site and metabolic mechanisms in *Aedes aegypti* and *Aedes albopictus* from Lahore, Pakistan. Sci. Rep..

[2] Smith LB, Kasai S, Scott JG (2016). Pyrethroid resistance in *Aedes aegypti* and *Aedes albopictus*: Important mosquito vectors of human diseases. Pestic. Biochem. Physiol..

[3] Zhang Y (2023). Resistance to three pyrethroid insecticides and knockdown resistance gene mutations in *Aedes albopictus* in Guiyang, Guizhou Province, China. Chin. J. Vect. Biol. Control.

[4] Wilson AL (2020). The importance of vector control for the control and elimination of vector-borne diseases. PLoS Negl. Trop. Dis..

[5] Grung M (2015). Pesticide levels and environmental risk in aquatic environments in China—A review. Environ. Int..

[6] Wang Y (2016). A survey of insecticide resistance in *Aedes albopictus* (Diptera: Culicidae) during a 2014 dengue fever outbreak in Guangzhou, China. J. Econ. Entomol..

[7] Ai LY, Hou J, Chen EF (2017). Review on control techniques of *Aedes mosquitoes*, important vectors of the communicable diseases. Chin. J. Vect. Biol. Control.

[8] Lu X (2013). Chikungunya emergency in China: Microevolution and genetic analysis for a local outbreak. Virus Gene.

[9] Gao JP, Chen HM, Shi H, Peng H, Ma Y-J (2018). Correlation between adult pyrethroid resistance and knockdown resistance (kdr) mutations in
* Aedes albopictus
* (Diptera: Culicidae) field populations in China. Infect. Dis. Poverty.

[10] Moyes CL (2017). Contemporary status of insecticide resistance in the major *Aedes* vectors of arboviruses infecting humans. PLoS Negl. Trop. Dis..

[11] Wang YY, Jiang ZK (2016). Development and application of public health pesticides in China, 2013–2016. Chin. J. Vect. Biol. Control.

[12] Zhao C (2023). Knockdown resistance mutations distribution and characteristics of *Aedes albopictus* field populations within eleven dengue local epidemic provinces in China. Front. Cell. Infect. Microbiol..

[13] Wei Y (2021). Insecticide susceptibility status and knockdown resistance (kdr) mutation in
* Aedes albopictus
* in China. Parasites Vectors.

[14] Quélennec G (1988). Pyrethroids in the WHO pesticide evaluation scheme (WHOPES). Parasitol. Today.

[15] Xu J (2018). Comparative transcriptome analysis and RNA interference reveal CYP6A8 and SNPs related to pyrethroid resistance in *Aedes albopictus*. PLoS Negl. Trop. Dis..

[16] Hirata K (2014). A single crossing-over event in voltage-sensitive Na+ channel genes may cause critical failure of dengue mosquito control by insecticides. PLoS Negl. Trop. Dis..

[17] Soderlund DM (2011). Molecular mechanisms of pyrethroid insecticide neurotoxicity: Recent advances. Arch. Toxicol..

[18] Zheng X, Cai W, Xu X, Jia Z, Wei Y (2020). Preliminary selection and analysis of deltamethrin-resistant strains of *Aedes albopictus* in the laboratory. Vector Borne Zoonotic Dis..

[19] Brengues C (2003). Pyrethroid and DDT cross-resistance in *Aedes aegypti* is correlated with novel mutations in the voltage-gated sodium channel gene. Med. Vet. Entomol..

[20] Meng FX, Wang YG, Feng L, Liu QY (2015). Review on dengue prevention and control and integrated mosquito management in China. Chin. J. Vector Biol. Control.

[21] Liu H-M (2012). Identification of TCT, a novel knockdown resistance allele mutation and analysis of resistance detection methods in the voltage-gated Na+ channel of *Culex pipiens pallens* from Shandong Province, China. Mol. Med. Rep..

[22] Smith LB, Kasai S, Scott JG (2017). Voltage-sensitive sodium channel mutations S989P + V1016G in *Aedes aegypti* confer variable resistance to pyrethroids, DDT and oxadiazines. Pest. Manag. Sci..

[23] Kushwah RBS, Dykes CL, Kapoor N, Adak T, Singh OP (2015). Pyrethroid-resistance and presence of two knockdown resistance (kdr) mutations, F1534C and a novel mutation T1520I, in Indian
*Aedes aegypti*. PLoS Negl. Trop. Dis..

[24] Kasai S (2011). First detection of a putative knockdown resistance gene in major mosquito vector, Aedes albopictus. Jpn. J. Infect. Dis..

[25] Chen H (2016). First identification of
kdr
allele F1534S in VGSC gene and its association with resistance to pyrethroid insecticides in
* Aedes albopictus
* populations from Haikou City, Hainan Island, China. Infect. Dis. Poverty.

[26] Zhou X (2019). Knockdown resistance (kdr) mutations within seventeen field populations of
* Aedes albopictus
* from Beijing China: First report of a novel V1016G mutation and evolutionary origins of *kdr* haplotypes. Parasites Vectors.

[27] Tancredi A (2020). Tracing temporal and geographic distribution of resistance to pyrethroids in the arboviral vector *Aedes albopictus*. PLoS Negl. Trop. Dis..

[28] Xu J (2016). Multi-country survey revealed prevalent and novel F1534S mutation in voltage-gated sodium channel (VGSC) gene in
*Aedes albopictus*. PLoS Negl. Trop. Dis..

[29] Kasai S (2019). First detection of a Vssc allele V1016G conferring a high level of insecticide resistance in *Aedes albopictus* collected from Europe (Italy) and Asia (Vietnam), 2016: A new emerging threat to controlling arboviral diseases. Eurosurveillance.

[30] Wang X (2015). Resistance to pyrethroid insecticides and analysis of knockdown resistance (*kdr* ) gene mutations in *Aedes albopictus* from Haikou City. Acad. J. Sec. Mil. Med. Univ..

[31] Campbell C (1997). Public health efforts in China before 1949 and their effects on mortality: The case of Beijing. Soc. Sci. Hist..

[32] Hou J (2020). Insecticide resistance of *Aedes albopictus* in Zhejiang Province, China. BST.

[33] Li Y (2020). Widespread multiple insecticide resistance in the major dengue vector *Aedes albopictus* in Hainan Province, China. Pest. Manag. Sci..

[34] Wu Y (2021). Knockdown resistance (kdr) mutations I1532T and F1534S were identified in
* Aedes albopictus
* field populations in Zhejiang Province, Central China. Front. Cell. Infect. Microbiol..

[35] Ravula AR, Yenugu S (2021). Pyrethroid based pesticides—chemical and biological aspects. Crit. Rev. Toxicol..

[36] Chareonviriyahpap T, Aum-aung B, Ratanatham S (1999). Current insecticide resistance patterns in mosquito vectors in Thailand. Southeast Asian J. Trop. Med. Public Health.

[37] Liang WQ (2018). Resistance of *Aedes albopictus* to commonly used insecticides in Guiyang City of China. Chin. J. Hyg. Insect. Equip..

[38] Wang D (2021). Monitoring and analysis of insecticide resistance of *Aedes albopictus* in Xingyi and Chishui cities of Guizhou Province, China. Chin. J. Vector Biol. Control.

[39] Li Y (2018). Evidence for multiple-insecticide resistance in urban *Aedes albopictus* populations in southern China. Parasites Vectors.

[40] Yang X, Zhou Y, Sun Y, Liu J, Jiang D (2021). Multiple insecticide resistance and associated mechanisms to volatile pyrethroid in an *Aedes albopictus* population collected in southern China. Pestic. Biochem. Physiol..

[41] Yue Y, Liu X, Xu M, Ren D, Liu Q (2019). Epidemiological dynamics of dengue fever in mainland China, 2014–2018. Int. J. Infect. Dis..

[42] Lin H (2020). Epidemiological characteristics of dengue in mainland China from 1990 to 2019. Medicine (Madr.).

[43] Messina JP (2019). The current and future global distribution and population at risk of dengue. Nat. Microbiol..

[44] Du M, Jing W, Liu M, Liu J (2021). The global trends and regional differences in incidence of dengue infection from 1990 to 2019: An analysis from the global burden of disease study 2019. Infect. Dis. Ther..

[45] Liu H (2020). Bionomics and insecticide resistance of *Aedes albopictus* in Shandong, a high latitude and high-risk dengue transmission area in China. Parasites Vectors.

[46] Ishak IH, Jaal Z, Ranson H, Wondji CS (2015). Contrasting patterns of insecticide resistance and knockdown resistance (kdr) in the dengue vectors
*Aedes aegypti* and
* Aedes albopictus
* from Malaysia. Parasites Vectors.

[47] Khan HAA (2020). Resistance to insecticides and synergism by enzyme inhibitors in *Aedes albopictus* from Punjab, Pakistan. Sci. Rep..

[48] Chen H (2021). The pattern of kdr mutations correlated with the temperature in field populations of
*Aedes albopictus* in China. Parasites Vectors.

[49] Lu, B. L. *et al.* Insecta: Diptera. History of animals in China (ed. Zhu, H.F.) (Science Press, 1997).

[50] Organization, W. H. Guidelines for laboratory and field testing of mosquito larvicides (2005).

[51] Organization, W. H. Test procedures for insecticide resistance monitoring in malaria vector mosquitoes (2016).

